# Testosterone deficiency reduces the effects of late cardiac remodeling after acute myocardial infarction in rats

**DOI:** 10.1371/journal.pone.0213351

**Published:** 2019-03-21

**Authors:** Rafaela de Araujo Fernandes Corrêa, Eduardo Hertel Ribeiro, Sara Bianca Oliveira Mendes, Priscila Mendonça dos Santos, Miracle Vitória Albino da Silva, Daniel Ferron Silva, Igor Peixoto Biral, Priscila Rossi de Batista, Dalton Valentim Vassallo, Athelson Stefanon Bittencourt, Ivanita Stefanon, Aurélia Araújo Fernandes

**Affiliations:** 1 Department of Morphology, Federal University of Espírito Santo, Vitória, ES, Brazil; 2 Department of Physiological Sciences, Federal University of Espírito Santo, Vitória, ES, Brazil; 3 Department of Physiotherapy, School of Sciences Santa Casa de Misericórdia de Vitória, Vitória, ES, Brazil; Max Delbruck Centrum fur Molekulare Medizin Berlin Buch, GERMANY

## Abstract

Testosterone is associated with an increased risk of coronary heart disease. This study evaluated cardiac remodeling 60 days after myocardial infarction (MI) in rats with testosterone deficiency. One week after castration, the animals underwent myocardial infarction. Rats were divided into four groups: orchidectomized (OCT); orchidectomized and infarcted (OCT+MI), MI and control (Sham). The myocyte cross-sectional area and the papillary muscle contractility were evaluated 8 weeks after MI. The coronary bed was perfused with Biodur E20 resin to evaluate the neovascularization after MI. Data were expressed as mean ± SEM followed by ANOVA. Castration reduced myocyte hypertrophy when compared to Sham and myocardial infarction alone as well as preserved the contraction force and activation time after myocardial infarction. After beta-adrenergic stimulation, activation and relaxation kinetics were less impaired in the OCT+MI group than in the MI group. Contraction force was preserved in the OCT+MI group after beta-adrenergic stimulation. Multiple scanning electronic microscope images were obtained to characterize changes in the coronary arteries. Capillary density index was increased in the MI and OCT+MI groups compared with control. The MI and OCT+MI groups were characterized by irregular vessel arrangements with distorted shape, abrupt changes in vessel direction, as well as abrupt changes in diameter after bifurcations when compared to Sham and OCT. The results indicated that testosterone deficiency attenuates adverse cardiac remodeling after MI. Novel findings in this study were that testosterone deficiency in rats, induced by castration, changes the later remodeling after MI, when compared with non castrated rats. The absence of this androgenous hormone seems to be benefic against pathological hypertrophy.

## Introduction

Testosterone is the main androgen of male circulation. It is responsible for the development and maintenance of sexual characteristics in males and the anabolic state of tissues [1]. The androgen level reaches a peak in men around the age of 30, after which, testosterone levels decrease at a rate of 1–2% per year. In the last two decades there has been a significant increase in the number of prescriptions for testosterone replacement therapy in elderly men [2]. However, there are few data about the risks or benefits to the cardiovascular system of hormone replacement in men with a decline in age-related androgen levels [3]. The American Society of Endocrinology and the Food and Drug Administration (FDA) have issued reports about the potential increased cardiovascular risks associated with testosterone replacement therapy and encouraged further investigations in this area [4]. It is known that testosterone exerts action on the cardiovascular system through androgen receptors present in the endothelium, vascular smooth muscle and cardiomyocytes [3]. This androgen can adversely affect the cardiovascular system by decreasing HDL-cholesterol, as well as contributing to thrombogenicity by regulating the expression of thromboxane A2 receptors [5]. Among the cardiovascular diseases, acute myocardial infarction (MI) stands out as an important cause of mortality and morbidity worldwide [6], mostly occurring in men after age 50, the age when there is a decrease in levels of male sex hormones [7].

This study aims to investigate the involvement of cardiac function after MI in castrated male rats, condition that mimics the period of andropause. We sought to identify which functional differences, histological and coronary angioarchitecture occur during late remodeling of the remaining tissue, two months after MI in orchidectomized and non-orchidectomized rats. We wanted to identify histological differences and kinetics of activation and relaxation after MI. Our results suggest that orchidectomy had a protective role on cardiac remodeling through the reduction in myocyte hypertrophy and improvement in cardiac contractility.

## Materials and methods

### Experimental design, animals and procedures

Male Wistar rats at 8 weeks of age (260–280 g) were used. The experimental protocols were approved by the Ethics Committee on Animal Use of the Federal University of Espírito Santo (15/2016). The animals were randomly assigned to four experimental groups: infarcted (MI), orchidectomized (OCT), orchidectomized and infarcted (OCT+MI) and sham infarction (Sham). The experiments were performed 2 months after infarction in order to analyze the cardiac remodeling in the late stage of its evolution [8] [9]. Within this 2-month interval, the animals were weighed every 15 days in order to verify whether there was any difference in weight gain between the groups. After hemodynamic and ponderal evaluations, the rats were then sacrificed, and the hearts were submitted to histological analysis, contractile papillary muscles evaluation and vascular molding by corrosion. The corrosion casts were subsequently analyzed by scanning electron microscopy (SEM). Orchidectomy was performed according to previously described methods [10]. Briefly, the animals were anesthetized intramuscularly with ketamine (50 mg/kg) and xylazine (10 mg/kg). A small incision was made at the posterior end of each scrotum to expose the testis by compression. The spermatic funiculum was tied at the height of the vas deferens, the testicles were removed and the scrotum sutured. The animals of the MI and OCT + MI groups underwent anterior interventricular coronary artery occlusion surgery for infarction induction. Briefly, the animals were anesthetized with a mixture of Ketamine (50 mg/kg) and Xylazine (10 mg/kg) intraperitoneally. Then, the left hemithorax trichotomy and thoracotomy was performed. The intercostal and pectoral muscles were divulsed, the ribs separated and the heart gently externalized by lateral compression of the chest. The aorta was then attached between the border of the left atrium and the sulcus of the pulmonary artery, approximately 3 millimeters distal to its origin, through the use of 6.0 mononylon wire. The heart was then repositioned in the thoracic cavity and the chest was closed. The surgical procedure of the infarction after opening of the thorax lasted at most 30 seconds [11]. The sham group underwent the same surgical procedure, except for the coronary artery ligation.

In our study was used about 200 rats on total. Our mortality indices after MI was 50%, corroborating with data previous, that showed mortality within the first 24 h after surgery is usually 40 to 60% [12].

The hemodynamic evaluation was realized two months after infarction induction. After, the animals were sacrificed, hearts and lungs were weighed. The lungs were dried for 48 hours. The water content of the lungs, the ratio between lung weight and body weight (LW/BW) and the ratio of heart weight to body weight (HW/BW) were calculated [8].

### Hemodynamic evaluation

Hemodynamic measurements were performed on all animals after intraperitoneal urethane (1.2 g / kg) anesthesia. Briefly, the right common carotid artery was catheterized with polyethylene catheter (PE50). The catheter was connected to a pressure transducer coupled to a data acquisition system (MP 100 Byopac Systems, Inc. CA). After recording the blood pressure (BP) and heart rate (HR), the catheter was advanced to the left ventricle to obtain the following measures: left ventricular systolic pressure, final diastolic pressure and positive and negative pressure derivatives (dP / dt ±). The animal was then sacrificed by cervical dislocation.

### Histological evaluation

Histological slides stained with Picrosirius red were used to calculate the infarct area and to quantify the percentage of interstitial collagen in the LV.

Infarct size was quantified histologically by planimetry. The LV was cut into three transverse sections: apex, middle ring (3 mm), and base. From the middle ring, 5 μm sections were cut at 100 μm intervals and stained with picrosirius red. Infarct size (fraction of the infarcted LV) was calculated as the average of all slices and expressed as a percentage of length [13]. Only rats with extensive infarcts (45%) were included in the study.

HE stained slides were used to calculate the cross-sectional area of the myocytes in the LV. All analyzes were done by the same observer. The sections were photographed using a 40x objective in a Zeiss optical microscope (Primo Star model) coupled to the Zeiss camera (model AxioCam ERc 5s). For quantification of collagen, 10 random fields per slide were photographed in the region remote from LV infarction, avoiding areas with large blood vessels. Through the Image J program, the percentage of reddish-colored image of Picrosirius red, corresponding to the area of the interstitial collagen [14], was evaluated in each photo. To evaluate the myocyte hypertrophy, the Image J program was used to measure the area of 50 myocytes positioned perpendicularly to the plane of the section and having both a visible nucleus and a clearly outlined and unbroken cell membrane in each animal [14].

### Papillary muscles contractility

Left ventricle (LV) muscle was dissected and placed in Krebs solution composed (mM) 120 NaCl, 5.4 KCl, 1.25 CaCl_2_, 1.2 MgCl_2_, 2 NaH_2_PO_4_, 1.2 Na_2_SO_4_, 18 NaHCO_3_ and 11 glucose. This solution was gassed with 5% CO_2_ and 95% O_2_ at a temperature of 30°C. The muscles were then fixed at their ends and stimulated by silver electrodes positioned parallel to their length. The stimulation lasted 10 ms using the voltage of about 8 V at a frequency of 0.5 Hz. After a stabilization period of 15 minutes, the muscles were stretched gradually until the developed strain reached the maximum level. The isometric force (g/g), time to peak (TTP) in ms, time to 50% of relaxation (TR50) in ms and maximum rate of tension development (+dF/dt max) and decline (−dF/dt max) were evaluated. After these initial measurements, the preparations were subjected to another stabilization period lasting 30 minutes for the evaluation of the positive inotropic effects due isoproterenol using 0.62 mM calcium in the bath. Isoproterenol at a dose of 1×10^−4^ M was added to the bath to evaluate the isometric force (g/g) and the maximum rate of tension development (+dF/dt max) and decline (−dF/dt max). Activation and relaxation kinetics were evaluated after to increasing isoproterenol concentrations (10^−7^ to 10^−2^ M).

### Vascular corrosion casts

The epoxy resin BIODUR E20 was used for corrosion vascular molding. After the hemodynamic procedure, thorax and abdomen were opened and the inferior vena cava was cut to drain the animal's blood. The aortic artery was tied about 1cm from the heart and the blood vessels connecting the heart and lungs were also attached. Subsequently, about 50 mL of heparinized saline (10 IU/mL) was injected through the polyethylene catheter in order to remove blood from the heart. About 3 mL of BIODUR E20 resin was slowly injected under manual pressure through the same catheter. The heart was removed and stored in water at room temperature for 48 hours for complete polymerization of the resin. Then, the hearts were immersed in 7% potassium hydroxide solution for 3 days for corrosion of all biological material. Finally, the casts were washed in a thin stream of water to remove any biological material still present and stored in 90% alcohol for analysis by SEM.

### Structural analysis

For scanning electron microscopy analysis, the casts were dehydrated through 3 baths of 1 hour each in absolute alcohol and dried for 3 days at 40°C. Subsequently, the samples were mounted on aluminum stubs using double-sided conductive carbon tape. Then the material was gold plated on the argon metallizer at 30 mA for 2 minutes. Finally, the scanning electron microscopy was performed with tungsten (W) filament operated at 20 kV and a working distance of 15 mm. Photomicrographs were obtained in the region near the anterior descending coronary artery, using magnification from 150 to 2500 X for the qualitative analysis of the samples. In this analysis, the characteristics of the three-dimensional architecture of the blood vessels were evaluated. Quantitative analysis of the vascular density index was also performed. For this analysis, 5 micrographs of each heart (N = 4 animals per group) were obtained in a total of 20 photos per group with an magnification of 500 X and the threshold tool was used in the computer program Image J to select a cut-off point for the levels of the images obtained in the scanning electron microscopy. Gray levels above this point were set to white (refer to the vascular area) and lower levels were set to black (refer to the avascular area), thus creating a binary image. The computer program calculates the proportion between the white area and the total area in the binary image, thus providing the ratio between the vascular and avascular area in the image, which corresponds to the vascular density index [15] [16].

### Statistical analysis

The data are reported as the mean ± standard error of the mean (SEM). A two-way ANOVA was used to analyze the differences between groups, followed by the Tukey *post-hoc*. The comparison between the infarct areas was performed through the unpaired Student's t-test. A p < 0.05 was considered significant. The statistical program GraphPad Prism 6.0 was used to analyze the data.

## Results

### Body weight

**Fig 1:**
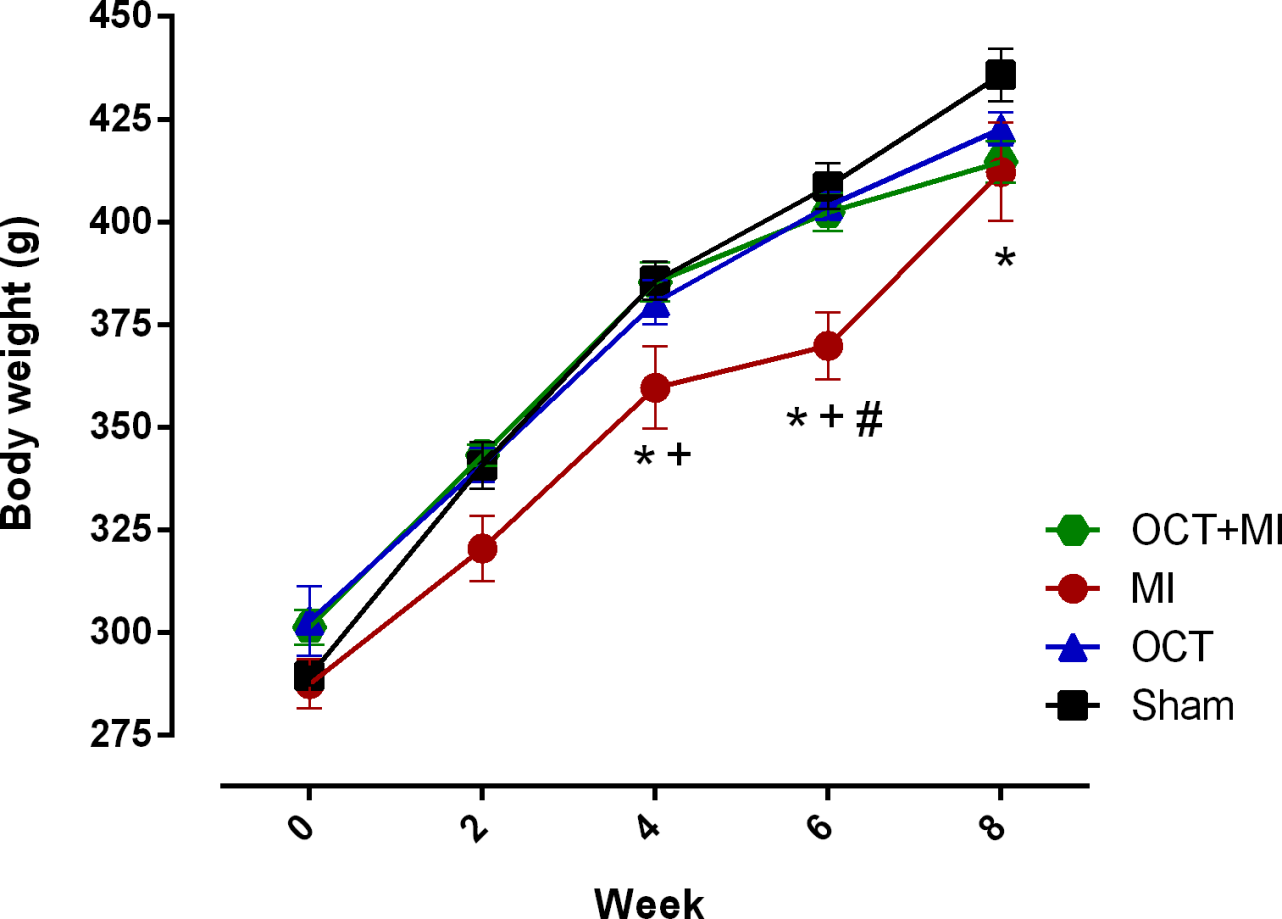
Changes in body weight of animals over time (Week). Data represented as mean ± SEM. For statistical analysis, two-way ANOVA was used for repeated measurements followed by Tukey *post hoc*. * p <0.05 versus Sham; # p <0.05 versus OCT; + p <0.05 versus OCT+MI.

Weight loss is a characteristic of advanced chronic heart failure [17]. Fig 1 and S1 Table shows weight gain during 8 weeks following myocardial infarction. Myocardial infarction induced weight loss that was statistically significant after 4 weeks. Testosterone deficiency prevented weight loss after myocardial infarction during the entire treatment.

### Ponderal data

The HW/BW ratio was higher in the infarcted groups. The LW/BW ratio (Table 1) was higher in MI group, but there weren’t to sham in the OCT+MI group. These are indicative parameters of cardiac and pulmonary hypertrophy, respectively [18].

**Table 1:** Ponderal data.

	Sham (N = 10)	OCT (N = 10)	MI (N = 10)	OCT+MI (N = 10)
Body weight (g)	450 ± 8	422 ± 5	447 ± 8	437 ± 7
HW/BW (mg/g)	3.91 ± 0,17	3.88 ± 0,18	5.24 ± 0,36*^#^	5.47 ± 0.32*^#^
LW/BW (mg/g)	3.89 ± 0.19	4.01 ± 0.13	5.19 ± 0.29*^#^	4.81 ± 0.29
Lung Water (%)	77.52 ± 0.23	78.41 ± 0.33	78.26 ± 0.21	78.4 ± 0.25

HW/BW = heart-to-body weight; LW/BW = lung-to-body weight.

* p <0.05 versus Sham

# p < 0.05 versus OCT.

Differences were analyzed using a Two-way ANOVA followed by a Tukey *post hoc* test.

### Hemodynamic data

There was a significant increase in diastolic blood pressure in the MI and OCT+MI groups when compared to the sham group. This difference may be related to the compensatory activation of the sympathetic nervous system that occurs after myocardial infarction [19]. As expected, LVEDP was higher after myocardial infarction and it was not affected by testosterone deficiency. This increase in LVEDP is a consequence of the depression of the left ventricular performance that occurs after large infarctions [12] (Table 2).

**Table 2:** Hemodynamic data.

	Sham (N = 10)	OCT (N = 10)	MI (N = 10)	OCT+MI (N = 10)
SBP (mmHg)	96± 3	97 ± 4	102 ± 3	97 ± 4
DBP (mmHg)	54 ± 3	61 ± 3	67 ± 4*	66 ± 2*
MBP (mmHg)	68 ± 4	77 ± 3	81 ± 4*	80 ± 3*
HR (bpm)	321 ± 14	296 ± 6	314 ± 15	271 ± 16*
LVSP (mmHg)	102 ± 4	101 ± 5	107 ± 3	101 ± 5
LVEDP (mmHg)	3.7 ± 0.4	4.9 ± 0.2	7.9 ± 0.4*^#^	7.7 ± 0.5*^#^
+dP/dt (mmHg/s)	5790 ± 409	5954 ± 360	5089 ± 377	5358 ± 453
-dP/dt (mmHg/s)	-4044 ± 285	-4383 ± 301	-3552 ± 256	-3799 ± 292

SBP: systolic blood pressure; DBP: diastolic blood pressure, MBP: mean blood pressure; HR: heart rate, LVSP: left ventricle systolic pressure; LVEDP: left ventricle end diastolic pressure; *+*dP/dt: positive first time derivative; -dP/dt: negative first time derivative.

* p < 0.05 versus Sham

# p < 0.05 versus OCT.

Results are expressed as the means ± SEM. Differences were analyzed using a Two-way ANOVA followed by a Tukey *post hoc* test.

### Structural analysis

**Fig 2:**
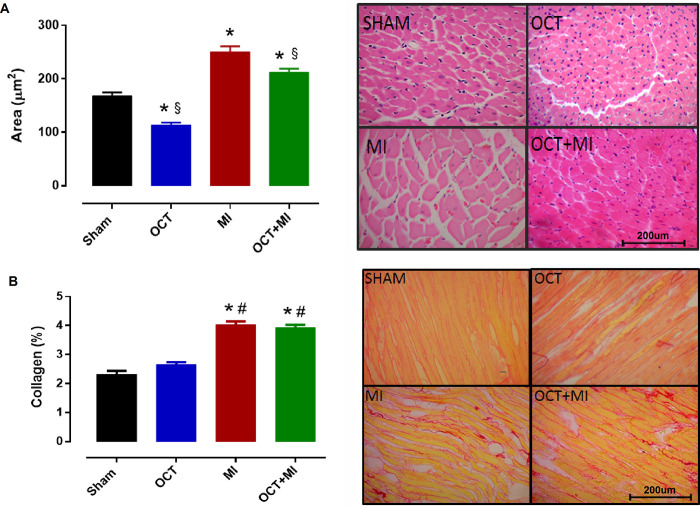
Histological evaluation in rat hearts. Myocyte cross-sectional area and representative images of the histological blades stained with HE (A) and percentage of interstitial collagen in the LV and representative images of the histological sections stained with Picrosirius red (B) from Sham, OCT, MI and OCT+MI. Objective: 40X. Scale bar: 200 μm. N = 10 animals per group. Data expressed as mean ± SEM. Statistical analysis: Two-way ANOVA followed by a Tukey *post hoc* test. * p < 0.05 vs Sham, # p < 0.05 vs OCT and §p < 0.05 vs MI.

The scar size in the MI group was 51.6 ± 1.9% and in the OCT+MI group, it was 47.8 ± 1.6%. There was no difference in infarct size between groups (S2 Table). The cross-sectional area of myocytes in the MI and OCT+MI groups was higher when compared to the sham and OCT groups (Fig 2A and S3 Table). In addition, this parameter was lower in the OCT+MI group as compared to the MI group, indicating that cardiac hypertrophy was smaller in castrated animals that underwent myocardial infarction as compared to myocardial infarction alone. Collagen deposition was increased in the MI and OCT+MI groups as compared to sham and OCT groups (Fig 2B and S3 Table).

### Contractility

**Fig 3:**
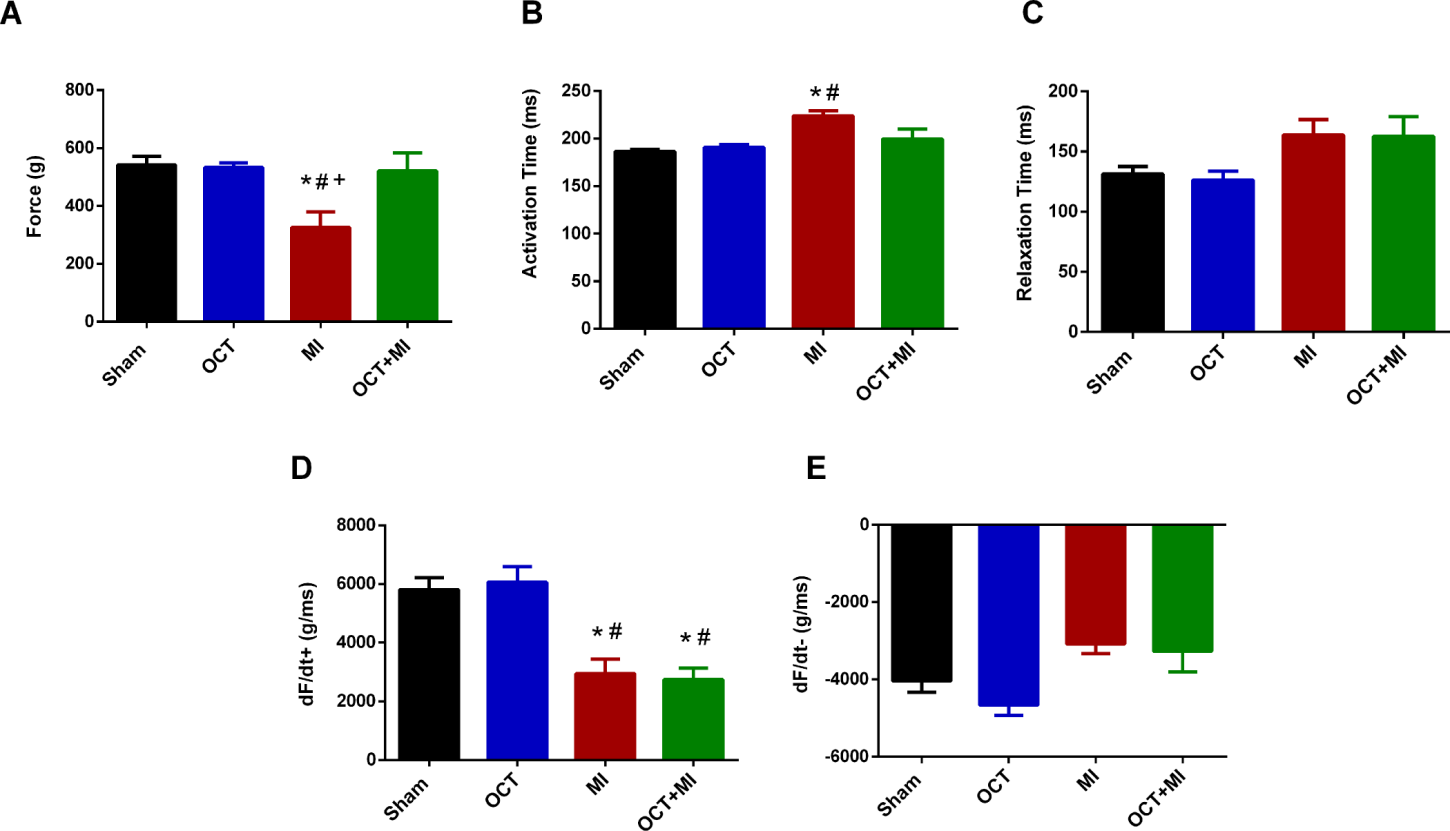
Contractility analyses. Isometric force (g / g) in the LV papillary muscles measure with 1.25 mM of calcium (3A), Activation Time (ms) (3B), Relaxation Time: TR50 (ms) (3C), maximum rate of force development (+dF/dt max, g/ms) in the LV papillary muscles (3D) and minimum rate of force development (-dF/dt g/ms) in the LV papillary muscles (3E). Groups: Sham (N = 4), OCT (N = 4), MI (N = 4) and OCT + MI groups (N = 4). Data expressed as mean ± SEM. The statistical analysis used was Two-way ANOVA followed by Tukey post hoc. * p < 0.05 vs Sham, # p < 0.05 vs OCT; + p < 0.05 versus OCT + MI.

Isometric contractility was preserved in the papillary muscles from the OCT and OCT+MI groups, but this response was impaired in the papillary muscles from the MI group (Fig 3A and S4 Table). The time to peak (TTP) was preserved in the OCT+MI group and impaired in the MI group (Fig 3B and S4 Table). There was no difference in relaxation time among groups (Fig 3C and S4 Table). The maximum rate of force development (+dF/dt max) was decreased in the MI and OCT+MI groups (Fig 3D and S4 Table). There was no difference in the minimum rate of force development (-dF/dt min) among groups (Fig 3E and S4 Table).

**Fig 4:**
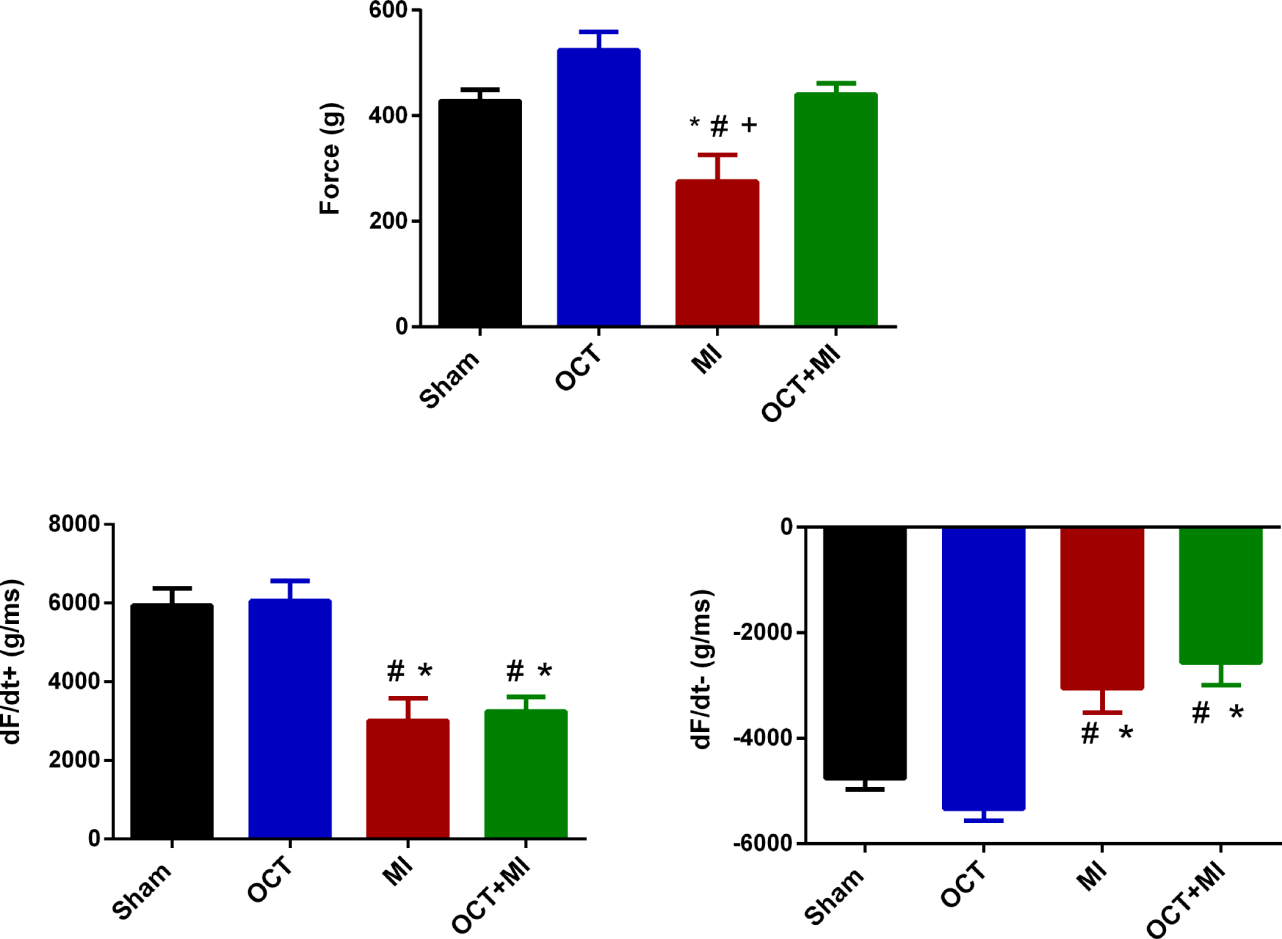
Contractility in response to isoproterenol 10^−4^ M. Isometric force (g/g) in LV papillary muscles (4A), maximum rate of force development (+dF/dt max) (4B) and minimum rate of force development (-dF/dt min) (4C). Groups: Sham (N = 4), OCT (N = 4), MI (N = 4) and OCT+MI (N = 4) groups. Data expressed as mean ± SEM. Statistical analysis: Two-way ANOVA followed by Tukey's post hoc. * p < 0.05 vs Sham; # p < 0.05 vs OCT.

Castration preserved the contraction force in response to isoproterenol, the same was not observed in the MI group (Fig 4A). The maximum and minimum rate of force development (+dF/dt and -dF/dt) were decreased in the MI and OCT+MI groups (Fig 4C and S5 Table).

**Fig 5:**
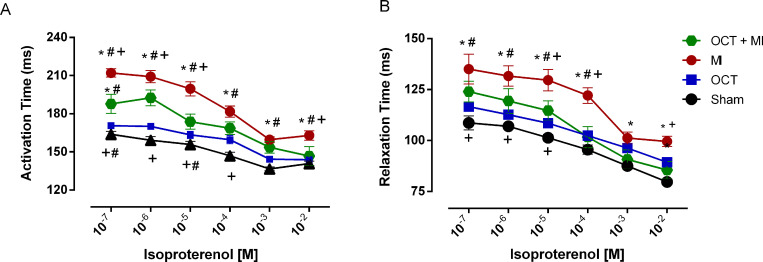
Activation Time (ms) (A) and Relaxation Time: RT50 (ms) (B) kinetic in LV papillary muscles in the presence of isoproterenol at concentrations 10^−7^ to 10^−2^ M. Sham (N = 6), OCT (N = 9), MI (N = 4) and OCT+MI (N = 4). Data expressed as mean ± SEM. Statistical analysis: 2-way ANOVA followed by Tukey *post hoc*. * p < 0.05 vs Sham; # p < 0.05 vs OCT; + p <0.05 versus OCT+MI.

The infarcted groups presented impaired activation kinetic throughout the dose-response curve to isoproterenol. However, the OCT+MI group had a better activation kinetic when compared to MI group (doses: 10^−7^, 10^−6^, 10^−5^ and 10^−2^ M) T (Fig 5A and S6 Table). The OCT+MI group had a better relaxation kinetic when compared to MI group (doses: 10^−5^, 10^−4^ and 10^−2^ M) (Fig 5B and S6 Table).

### Ultrastructural analysis

**Fig 6:**
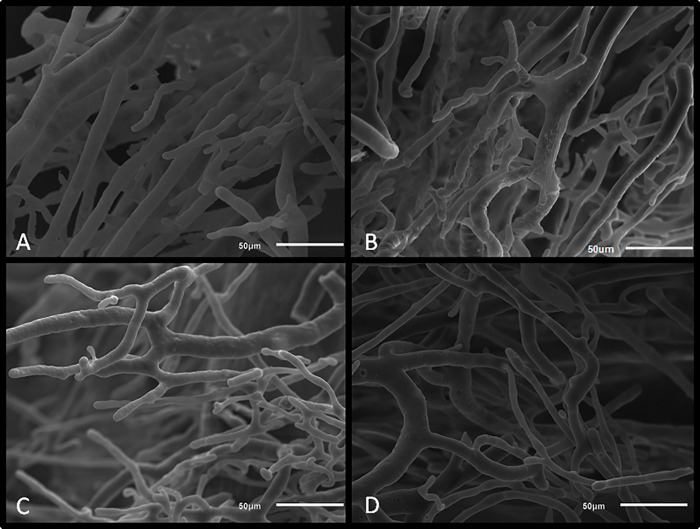
Scanning electron microscopy images of animal hearts. Groups: OCT (A and B) and Sham (C and D) groups. Scale bar: 50 μm.

**Fig 7:**
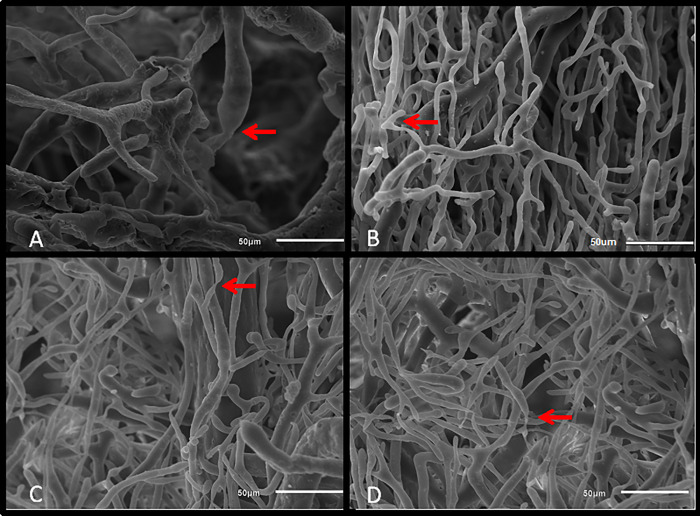
Scanning electron microscopy images of corrosion casts. Groups: MI (A and B) and OCT+MI groups (C and D). Arrows indicate places of abrupt narrowing of blood vessels. Scale bar: 50 μm.

Sham and OCT groups showed uniform vascular arrangements with linear orientation, small changes in vessel diameter after branching, as well as mild changes in vessel directions (Fig 6). In contrast, MI and OCT+MI groups were characterized by irregular vessel configurations with distorted shape, abrupt changes in vessel direction, and abrupt changes in diameter after bifurcations (Fig 7).

**Fig 8:**
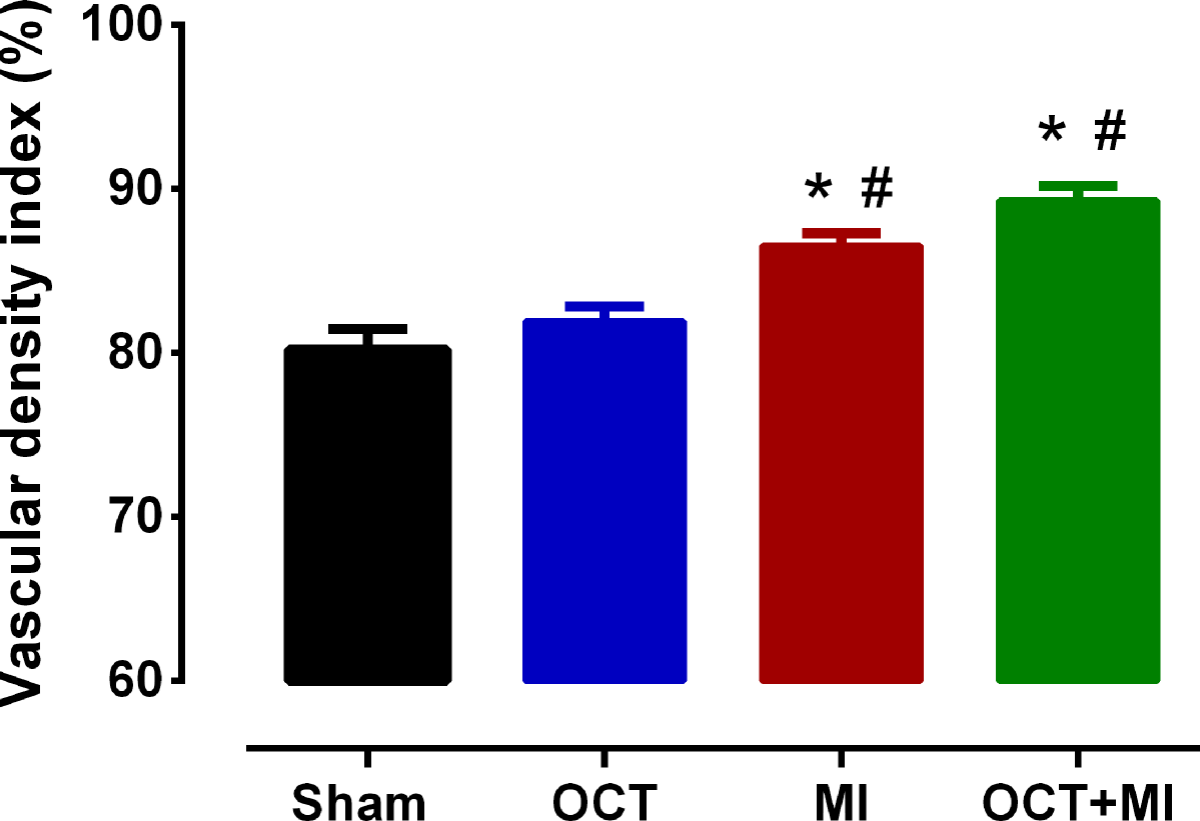
Vascular density index. Groups: Sham (N = 20), OCT (N = 20), MI N = 20) and OCT+MI groups (N = 20). Data expressed as mean ± SEM. The statistical analysis used was Two-way ANOVA followed by Tukey post hoc. * p < 0.05 vs Sham, # p < 0.05 vs OCT. For the analysis 20 photos were used per group.

The vascular density increased in the infarcted groups relative to the controls (Fig 8 and S7 Table).

## Discussion

Our results demonstrate that 2 months after MI, orchidectomized rats had a lower cross-sectional area of the LV myocytes when compared to the myocardial infarction alone as well as better contractility parameters.

The incidence of cardiovascular disease (CVD) and its severity differs between women and men [20]. The high levels of CVD in men could be related to the high levels of testosterone due to its pro-atherogenic and / or the absence of the protective effect of estrogens. Thus, it has been suggested that high levels of testosterone found in men would be deleterious to the cardiovascular system [21]. However, to date, the role of androgens in the heart remains controversial [22].

In this study, the OCT+MI group maintained the weight gain as compared to the Sham group. It is suggested that testosterone deficiency prevented significant weight loss after MI. It is known that men undergoing androgen deprivation therapy present weight gain that may reach 2.4% in the first year of treatment [23]. Testosterone exerts an important influence on the metabolism of adipose tissue. The activity of lipoprotein lipase, the main enzyme regulator of the uptake of triglycerides in adipose tissue, is inhibited by T, whereas lipid mobilization is stimulated [24], which may accentuate weight loss. It is known that weight change after MI can affect disease trajectory and weight loss greater than 5% was associated with a 70% increase in the risk of all-cause mortality and a similar increase in cardiovascular mortality in humans [25].

The cross-sectional area of myocytes was higher in the infarcted groups. This increase occurs due to post-MI ventricular remodeling. Hypertrophy is an adaptive response during post-infarct remodeling [26]. Functional myocardial loss after myocardial infarction results in a reactive hypertrophic adaptation of viable myocytes [27]. The area in OCT group was decreased compared to sham. This result demonstrates that gonadectomy in male rats can results in minor myocyte size due to alterations in cellular structure and cardiac hypertrophy. In future studies we will investigate this effect.

The cross-sectional area of LV myocytes was lower in the OCT+MI group when compared to the MI group, which could indicate that testosterone may affect cardiac hypertrophy. A study showed that orchidectomized and infarcted mice had a smaller cross-sectional area of myocytes when compared to infarcted males, confirming the role of testosterone in the induction of post-MI maladaptive hypertrophy [28]. This testosterone effect can be explained by the hypertrophic stimulation via the androgen receptor. The androgen receptor gene is expressed in cardiac myocytes and the androgens can mediate a significant hypertrophic response directly in these cells [29]. There was an increase in collagen deposition in all infarcted groups. The injury caused by MI results in the development of an extracellular matrix rich scar [30], but orchidectomy did not affect this parameter.

The isometric force (g/g) and TTP were preserved in the OCT+MI group and reduced in the MI group indicating that testosterone deficiency reduced contractility impairment after injury. After beta-adrenergic stimulation, the isometric force (g/g) in the OCT+MI group was similar to the controls. Activation and relaxation kinetics in the presence of isoproterenol at concentrations 10^−7^ to 10^−2^ M were better in the absence of testosterone after MI. These results suggest that testosterone deficiency contributed to the improvement of post-MI contraction force against beta-adrenergic agonist. Thus, it is possible to suggest that testosterone deficiency contributes to improved contractile response after MI. It is known that the cell changes induced by testosterone deficiency prevent impaired contractility caused by MI and increased transient calcium 8 weeks after the onset of the event [10]. This same study found an increase in SERCA expression in the left ventricle of testosterone-deficient infarcted animals, as well as increased SERCA / phospholamban ratio and phospholamban phosphorylation. It is known that in hearts with heart failure the function of SERCA is reduced [31], therefore, increases in the expression of this protein in castrated and infarcted rats as well as increased phospholamban phosphorylation may help explain the better parameters of contractility in the OCT+MI group in relation to MI.

The deleterious effects of testosterone after MI may be due to induction of hypertrophy via androgen receptors [29] and / or increasing apoptosis after MI, since a study found that in patients with heart failure post-MI, the rate of myocyte necrosis and apoptosis was higher in men than in women, which was associated with earlier onset of the disease in males [32].

Another aspect of the present study was the anatomical evaluation of the coronary bed in the groups of animals through the vascular corrosion casts associated with SEM. The vasculature of the heart ensures the metabolic and structural homeostasis of this organ. The blood vessels provide proper perfusion and are crucial for the growth and survival of cardiomyocytes. Inadequate perfusion of cardiac muscle may contribute to irreversible myocardial hibernation and decreased contractile function [33]. In the qualitative evaluation, we differentiate the groups regarding the morphological characteristics of the blood vessels. Sham and OCT groups were characterized by uniform vascular arrangements with linear orientation, small changes in vessel diameter after the ramifications, as well as mild changes in vessel directions. In contrast, the MI and OCT+MI groups were characterized by irregular vessel configurations with distorted shape, abrupt changes in vessel direction, as well as abrupt changes in diameter after bifurcations, changes already described in the literature as characteristics of the coronary bed of animals with heart failure [34, 35].

In the quantitative analysis, the vascular density index was increased in the MI and OCT+MI groups when compared to the sham and OCT groups. Chen et al., 2017 [35] also found increased capillary density in the myocardium of rats submitted to myocardial ischemia / reperfusion protocol when compared to control rats. An imbalance in the growth of blood vessels contributes to the pathogenesis of numerous disorders [36]. The vascular proliferation induced by hypertrophy and ischemia contributes to the angiogenesis of the capillaries. However, the consequences of vascular remodeling are detrimental in hearts with heart failure. In addition to the increase in the number of capillaries, there is also a change in their functional structure [35]. The similarity in vascular density index between the MI and OCT+MI groups may indicate that testosterone did not contribute significantly to the post-MI angiogenesis process.

Novel findings in this study were that testosterone deficiency in rats, induced by orchidectomy, changes the later remodeling after MI, when compared with non castrated rats. The absence of this androgynous hormone seems to be benefic against pathological hypertrophy.

## Conclusion

Testosterone deficiency, induced by orchidectomy in rats, altered the pattern of late remodeling post-infarction. The deficiency of this androgen resulted in a lower degree of pathological cardiac hypertrophy and an improvement in the contractile parameters of the LV papillary muscles, such as: contraction force (g / g) and shorter activation time, as well as a better contractile response to beta- (g / g) in the presence of 10^−4^ M isoproterenol and activation and relaxation kinetics in the dose-response curve ranging from 10^−7^ to 10^−2^ M of the same agonist. Orchidectomized animals did not present significant differences in the amount of interstitial collagen, vascular bed morphology and vascular density, compared to control animals, indicating that the main effect of testosterone on post-MI remodeling occurs in the myocyte, mainly through the induction of pathological hypertrophy and alteration of the contractile function.

## Supporting information

S1 Table Changes in body weight (g) of animals over time (Week). (DOCX)

S2 Table Infarct area (% of left ventricle). (DOCX)

S3 Table Histological evaluation in rat hearts. (DOCX)

S4 Table Contractility analyses of pappilary muscles. (DOCX)

S5 Table Contractility in response to isoproterenol 10^−4^ M. (DOCX)

S6 Table Activation Time (ms) and Relaxation Time (ms) in the LV papillary muscles in the presence of isoproterenol 10^−7^ to 10^−2^ M. (DOCX)

S7 Table Vascular density index. (DOCX)
