# Correction: Testosterone deficiency reduces the effects of late cardiac remodeling after acute myocardial infarction in rats

**DOI:** 10.1371/journal.pone.0226664

**Published:** 2019-12-12

**Authors:** Rafaela de Araujo Fernandes Corrêa, Rogério Faustino Ribeiro Júnior, Sara Bianca Oliveira Mendes, Priscila Mendonça dos Santos, Miracle Vitória Albino da Silva, Daniel Ferron Silva, Igor Peixoto Biral, Priscila Rossi de Batista, Dalton Valentim Vassallo, Athelson Stefanon Bittencourt, Ivanita Stefanon, Aurélia Araújo Fernandes

The Funding statement is incorrect. The correcting Funding statement is: This study was supported by FAPES- Espirito Santo Research and Innovation Support Foundation, Processos: 54690722/2011 and 84324600/2018; CNPq; UFES. The funders did not have any additional role in the study design, data collection and analysis, decision to publish, or preparation of the manuscript.
