# Supplementary material for: Testosterone deficiency reduces the effects of late cardiac remodeling after acute myocardial infarction in rats
Source: PLoS One. 2019 Mar 21;14(3):e0213351. doi: 10.1371/journal.pone.0213351 (PMC6428328; doi:10.1371/journal.pone.0213351)
Supplement: S1 Table — (DOCX) [file pone.0213351.s001.docx]

**S1Table. Changes in body weight (g) of animals over time (Week).**

| **WEEK** | **Sham** | | | | | | | | | |
| --- | --- | --- | --- | --- | --- | --- | --- | --- | --- | --- |
| 0 | 285.00 | 290.00 | 300.00 | 290.00 | 303.00 | 291.00 | 280.00 | 283.00 | 287.00 | 285.00 |
| 2 | 350.00 | 330.00 | 380.00 | 353.00 | 336.00 | 320.00 | 320.00 | 333.00 | 335.00 | 350.00 |
| 4 | 380.00 | 360.00 | 420.00 | 380.00 | 388.00 | 387.00 | 390.00 | 386.00 | 385.00 | 380.00 |
| 6 | 420.00 | 390.00 | 450.00 | 400.00 | 406.00 | 402.00 | 399.00 | 395.00 | 405.00 | 420.00 |
| 8 | 450.00 | 420.00 | 480.00 | 420.00 | 430.00 | 411.00 | 448.00 | 429.00 | 443.00 | 427.00 |
| **WEEK** | **OCT+MI** | | | | | | | | | |
| 0 | 300.00 | 320.00 | 308.00 | 284.00 | 320.00 | 292.00 | 306.00 | 303.00 | 300.00 | 280.00 |
| 2 | 327.00 | 340.00 | 350.00 | 343.00 | 351.00 | 348.00 | 340.00 | 347.00 | 352.00 | 334.00 |
| 4 | 371.00 | 400.00 | 387.00 | 370.00 | 410.00 | 380.00 | 385.00 | 400.00 | 387.00 | 363.00 |
| 6 | 401.00 | 408.00 | 389.00 | 396.00 | 420.00 | 410.00 | 400.00 | 422.00 | 407.00 | 371.00 |
| 8 | 410.00 | 418.00 | 404.00 | 402.00 | 426.00 | 418.00 | 410.00 | 432.00 | 440.00 | 386.00 |
| **WEEK** | **OCT** | | | | | | | | | |
| 0 | 290.00 | 293.00 | 295.00 | 280.00 | 290.00 | 352.00 | 280.00 | 283.00 | 345.00 | 320.00 |
| 2 | 330.00 | 333.00 | 343.00 | 333.00 | 347.00 | 355.00 | 338.00 | 326.00 | 369.00 | 334.00 |
| 4 | 366.00 | 370.00 | 392.00 | 365.00 | 390.00 | 406.00 | 380.00 | 356.00 | 405.00 | 374.00 |
| 6 | 397.00 | 388.00 | 417.00 | 405.00 | 416.00 | 408.00 | 411.00 | 390.50 | 412.00 | 395.00 |
| 8 | 410.50 | 412.00 | 436.00 | 427.00 | 428.00 | 417.00 | 438.00 | 424.00 | 434.00 | 400.00 |
| **WEEK** | **MI** | | | | | | | | | |
| 0 | 282.00 | 286.00 | 256.00 | 302.00 | 312.50 | 306.50 | 292.50 | 271.00 | 302.00 | 264.00 |
| 2 | 300.00 | 330.00 | 295.00 | 326.00 | 342.00 | 364.00 | 326.00 | 320.00 | 327.00 | 275.00 |
| 4 | 326.00 | 356.00 | 383.00 | 382.00 | 370.00 | 399.00 | 375.00 | 350.00 | 365.00 | 290.00 |
| 6 | 340.00 | 387.00 | 400.00 | 401.00 | 360.00 | 382.00 | 360.00 | 348.00 | 390.00 | 330.00 |
| 8 | 349.00 | 420.00 | 445.00 | 417.00 | 415.00 | 457.00 | 423.00 | 440.00 | 413.00 | 343.00 |
