# Supplementary material for: Testosterone deficiency reduces the effects of late cardiac remodeling after acute myocardial infarction in rats
Source: PLoS One. 2019 Mar 21;14(3):e0213351. doi: 10.1371/journal.pone.0213351 (PMC6428328; doi:10.1371/journal.pone.0213351)
Supplement: S2 Table — (DOCX) [file pone.0213351.s002.docx]

**S2 Table. Infarct area (% of left ventricle)**

| **INFARCT AREA** | |
| --- | --- |
| **MI** | **OCT+MI** |
| 52.126 | 47.062 |
| 46.114 | 41.144 |
| 45.321 | 55.420 |
| 43.682 | 41.669 |
| 50.672 | 49.068 |
| 45.741 | 55.503 |
| 57.884 | 50.815 |
| 58.765 | 45.492 |
| 58.200 | 43.368 |
| 57.397 | 48.954 |
