# Supplementary material for: Testosterone deficiency reduces the effects of late cardiac remodeling after acute myocardial infarction in rats
Source: PLoS One. 2019 Mar 21;14(3):e0213351. doi: 10.1371/journal.pone.0213351 (PMC6428328; doi:10.1371/journal.pone.0213351)
Supplement: S3 Table — (DOCX) [file pone.0213351.s003.docx]

**S3Table. Histological evaluation in rat hearts**

| **CARDIOMIOCYTE AREA (μm^2^)** | | | |
| --- | --- | --- | --- |
| **Sham** | **OCT** | **MI** | **OCT+MI** |
| 167.92 | 104.2 | 227.7584 | 221.5 |
| 144.1 | 103.6 | 242.9988 | 213.93 |
| 169.14 | 109 | 235.9798 | 216.42 |
| 165.71 | 107 | 301.4579 | 186.73 |
| 173.17 | 100.5 | 233.6048 | 228.45 |
| 190.95 | 130.55 | 197.8498 | 209.88 |
| 171.11 | 107.35 | 295.8314 | 254.51 |
| 163.33 | 144.1 | 275.3481 | 180.57 |
| 136.5 | 128.67 | 241.5264 | 212.88 |
| 202.49 | 98.75 | 255.936 | 199.52 |

| **LEFT VENTRICLE COLLAGEN (%)** | | | |
| --- | --- | --- | --- |
| **Sham** | **OCT** | **MI** | **OCT+MI** |
| 2.95 | 2.98 | 3.71 | 4.16 |
| 1.84 | 2.74 | 3.31 | 3.53 |
| 2.19 | 2.9 | 4.22 | 4.44 |
| 2.74 | 2.77 | 3.94 | 3.73 |
| 2.35 | 2.72 | 4.32 | 3.84 |
| 2.5 | 2.86 | 3.94 | 4.04 |
| 2.65 | 2.57 | 3.78 | 4.23 |
| 2.15 | 2.31 | 4.06 | 3.49 |
| 1.98 | 2.34 | 4.48 | 4.11 |
| 1.86 | 2.46 | 4.52 | 3.75 |
