# Supplementary material for: Testosterone deficiency reduces the effects of late cardiac remodeling after acute myocardial infarction in rats
Source: PLoS One. 2019 Mar 21;14(3):e0213351. doi: 10.1371/journal.pone.0213351 (PMC6428328; doi:10.1371/journal.pone.0213351)
Supplement: S4 Table — (DOCX) [file pone.0213351.s004.docx]

**S4 Table. Contractility analyses of pappilary muscles.**

| **FORCE (g/g)** | | | | | | |
| --- | --- | --- | --- | --- | --- | --- |
| **Sham** | | **OCT** | | **MI** | | **OCT+MI** |
| 621.0130 | | 563.1030 | | 291.7950 | | 432.959000 |
| 535.9430 | | 501.0640 | | 452.8570 | | 698.542000 |
| 527.5000 | | 512.6610 | | 359.7560 | | 513.131000 |
| 483.3720 | | 554.7370 | | 198.6550 | | 439.655000 |
|  | |  | |  | |  |
| **ACTIVATION TIME (ms)** | | | | | | |
| **Sham** | **OCT** | | **MI** | | **OCT+MI** | |
| 180 | 190 | | 230 | | 200 | |
| 190 | 190 | | 210 | | 180 | |
| 185 | 185 | | 235 | | 190 | |
| 190 | 198 | | 220 | | 228 | |

| **RELAXATION TIME (ms)** | | | |
| --- | --- | --- | --- |
| **Sham** | **OCT** | **MI** | **OCT+MI** |
| 115 | 130 | 155 | 120 |
| 130 | 120 | 200 | 170 |
| 145 | 145 | 160 | 160 |
| 135 | 110 | 140 | 200 |

| **dF/dt+ (g/ms)** | | | |
| --- | --- | --- | --- |
| **Sham** | **OCT** | **MI** | **OCT+MI** |
| 6801.7720 | 7366.176000 | 3703.6840 | 3773.8780 |
| 6054.4340 | 6483.288000 | 3116.3410 | 1933.5350 |
| 5468.2140 | 5172.241000 | 3435.6040 | 2876.8890 |
| 4915.2330 | 5196.842000 | 1549.2440 | 2381.0340 |

| **dF/dt – (g/ms)** | | | |
| --- | --- | --- | --- |
| **Sham** | **OCT** | **MI** | **OCT+MI** |
| -4823.0380 | -4398.9660 | -2871.3410 | -2837.3470 |
| -4050.7550 | -4758.5000 | -3435.2100 | -3711.0200 |
| -3426.1180 | -5372.5530 | -3529.6700 | -2039.6550 |
| -3864.0540 | -4138.1580 | -2502.6890 | -4508.8240 |
