# Supplementary material for: Testosterone deficiency reduces the effects of late cardiac remodeling after acute myocardial infarction in rats
Source: PLoS One. 2019 Mar 21;14(3):e0213351. doi: 10.1371/journal.pone.0213351 (PMC6428328; doi:10.1371/journal.pone.0213351)
Supplement: S5 Table — (DOCX) [file pone.0213351.s005.docx]

**S5 Table. Contractility** **in response to isoproterenol 10^-4^ M**

| **FORCE (g/g)** | | | |
| --- | --- | --- | --- |
| **Sham** | **OCT** | **MI** | **OCT+MI** |
| 478.8610 | 583.2350 | 264.1030 | 463.226000 |
| 449.2450 | 584.1100 | 399.4250 | 478.283000 |
| 396.1180 | 448.1910 | 285.2440 | 439.592000 |
| 386.3640 | 481.7020 | 152.2690 | 379.778000 |

| **dF/dt+ (g/ms)** | | | |
| --- | --- | --- | --- |
| **Sham** | **OCT** | **MI** | **OCT+MI** |
| 4763.3640 | 5598.816000 | 3255.1280 | 4220.6060 |
| 6729.7470 | 7230.000000 | 4272.7470 | 2744.4440 |
| 5799.8200 | 6501.918000 | 2980.0000 | 3384.5920 |
| 6464.7170 | 4863.534000 | 1522.8570 | 2631.8970 |

| **dF/dt- (g/ms)** | | | |
| --- | --- | --- | --- |
| **Sham** | **OCT** | **MI** | **OCT+MI** |
| -4364.860000 | -5587.750000 | -2808.8900 | -2109.080000 |
| -5320.000000 | -5040.860000 | -4251.6500 | -2087.780000 |
| -4527.060000 | -5837.400000 | -3106.7100 | -3843.270000 |
| -4815.570000 | -4890.260000 | -2038.4900 | -2211.550000 |
