# Supplementary material for: Testosterone deficiency reduces the effects of late cardiac remodeling after acute myocardial infarction in rats
Source: PLoS One. 2019 Mar 21;14(3):e0213351. doi: 10.1371/journal.pone.0213351 (PMC6428328; doi:10.1371/journal.pone.0213351)
Supplement: S6 Table — (DOCX) [file pone.0213351.s006.docx]

**S6 Table. Activation Time (ms) and Relaxation Time (ms) in the LV papillary muscles in the presence of isoproterenol 10^-7^ to 10^-2^ M**

**ACTIVATION TIME**

| **Dose** | **Sham** | **Sham** | **Sham** | **Sham** | **Sham** | **Sham** |  |  |  |
| --- | --- | --- | --- | --- | --- | --- | --- | --- | --- |
| **10-7** | 163 | 163 | 167 | 163 | 155 | 172 |  |  |  |
| **10-6** | 153 | 160 | 163 | 148 | 163 | 167 |  |  |  |
| **10-5** | 155 | 168 | 153 | 153 | 153 | 152 |  |  |  |
| **10-4** | 142 | 145 | 152 | 143 | 142 | 158 |  |  |  |
| **10-3** | 133 | 135 | 135 | 143 | 135 | 138 |  |  |  |
| **10-2** | 138 | 138 | 148 | 138 | 137 | 145 |  |  |  |
| **Dose** | **OCT** | **OCT** | **OCT** | **OCT** | **OCT** | **OCT** | **OCT** | **OCT** | **OCT** |
| **10-7** | 167 | 183 | 172 | 164 | 177 | 173 | 173 | 162 | 165 |
| **10-6** | 153 | 167 | 177 | 169 | 171 | 174 | 177 | 173 | 170 |
| **10-5** | 165 | 180 | 160 | 151 | 161 | 155 | 165 | 165 | 168 |
| **10-4** | 140 | 158 | 163 | 155 | 155 | 174 | 161 | 163 | 167 |
| **10-3** | 145 | 153 | 143 | 133 | 142 | 139 | 145 | 150 | 148 |
| **10-2** | 143 | 148 | 143 | 143 | 142 | 135 | 148 | 142 | 150 |
| **Dose** | **MI** | **MI** | **MI** | **MI** |  |  |  |  |  |
| **10-7** | 207 | 207 | 215 | 220 |  |  |  |  |  |
| **10-6** | 220 | 210 | 197 | 210 |  |  |  |  |  |
| **10-5** | 207 | 183 | 202 | 207 |  |  |  |  |  |
| **10-4** | 180 | 190 | 170 | 187 |  |  |  |  |  |
| **10-3** | 165 | 155 | 157 | 162 |  |  |  |  |  |
| **10-2** | 163 | 158 | 157 | 173 |  |  |  |  |  |
| **Dose** | **OCT+MI** | **OCT+MI** | **OCT+MI** | **OCT+MI** |  |  |  |  |  |
| **10-7** | 210 | 183 | 179 | 178 |  |  |  |  |  |
| **10-6** | 207 | 182 | 199 | 182 |  |  |  |  |  |
| **10-5** | 187 | 160 | 168 | 180 |  |  |  |  |  |
| **10-4** | 183 | 163 | 164 | 164 |  |  |  |  |  |
| **10-3** | 167 | 147 | 152 | 149 |  |  |  |  |  |
| **10-2** | 165 | 148 | 145 | 129 |  |  |  |  |  |

**RELAXATION TIME**

| **Dose** | **Sham** | **Sham** | **Sham** | | **Sham** | | | **Sham** | **Sham** |  |  |  |  |
| --- | --- | --- | --- | --- | --- | --- | --- | --- | --- | --- | --- | --- | --- |
| **10-7** | 97 | 112 | 108 | | 102 | | | 120 | 113 |  |  |  |  |
| **10-6** | 105 | 107 | 110 | | 103 | | | 107 | 110 |  |  |  |  |
| **10-5** | 95 | 103 | 98 | | 102 | | | 105 | 105 |  |  |  |  |
| **10-4** | 88 | 100 | 88 | | 98 | | | 98 | 100 |  |  |  |  |
| **10-3** | 85 | 83 | 92 | | 88 | | | 92 | 85 |  |  |  |  |
| **10-2** | 77 | 80 | 82 | | 82 | | | 83 | 75 |  |  |  |  |
| **Dose** | **OCT** | **OCT** | | **OCT** | | **OCT** | | **OCT** | **OCT** | **OCT** | **OCT** | **OCT** |  |
| **10-7** | 117 | 120 | | 120 | | 122 | | 106 | 120 | 116 | 108 | 120 |  |
| **10-6** | 112 | 107 | | 113 | | 109 | | 117 | 116 | 111 | 117 | 113 |  |
| **10-5** | 110 | 103 | | 115 | | 105 | | 110 | 111 | 114 | 102 | 107 |  |
| **10-4** | 85 | 105 | | 102 | | 103 | | 106 | 102 | 109 | 108 | 102 |  |
| **10-3** | 95 | 100 | | 88 | | 101 | | 95 | 92 | 103 | 100 | 92 |  |
| **10-2** | 90 | 95 | | 90 | | 85 | | 87 | 86 | 95 | 95 | 82 |  |
| **Dose** | **Infarto** | **Infarto** | **Infarto** | | **Infarto** | |  |  |  |  |  |  |  |
| **10-7** | 125 | 128 | 157 | | 130 | |  |  |  |  |  |  |  |
| **10-6** | 125 | 123 | 145 | | 133 | |  |  |  |  |  |  |  |
| **10-5** | 122 | 120 | 135 | | 142 | |  |  |  |  |  |  |  |
| **10-4** | 115 | 117 | 132 | | 125 | |  |  |  |  |  |  |  |
| **10-3** | 98 | 98 | 110 | | 98 | |  |  |  |  |  |  |  |
| **10-2** | 95 | 98 | 107 | | 98 | |  |  |  |  |  |  |  |
| **Dose** | **OCT+MI** | **OCT+MI** | **OCT+MI** | | **OCT+MI** | |  |  |  |  |  |  |  |
| **10-7** | 128 | 112 | 135 | | 121 | |  |  |  |  |  |  |  |
| **10-6** | 127 | 108 | 133 | | 110 | |  |  |  |  |  |  |  |
| **10-5** | 122 | 108 | 124 | | 105 | |  |  |  |  |  |  |  |
| **10-4** | 102 | 92 | 116 | | 97 | |  |  |  |  |  |  |  |
| **10-3** | 98 | 83 | 99 | | 82 | |  |  |  |  |  |  |  |
| **10-2** | 95 | 77 | 90 | | 80 | |  |  |  |  |  |  |  |
