# Supplementary material for: Testosterone deficiency reduces the effects of late cardiac remodeling after acute myocardial infarction in rats
Source: PLoS One. 2019 Mar 21;14(3):e0213351. doi: 10.1371/journal.pone.0213351 (PMC6428328; doi:10.1371/journal.pone.0213351)
Supplement: S7 Table — (DOCX) [file pone.0213351.s007.docx]

**S7 Table. Vascular density index**

| **Sham** | **OCT** | **MI** | **OCT+MI** |
| --- | --- | --- | --- |
| 75.15 | 81.27 | 83.60 | 89.63 |
| 85.02 | 85.45 | 84.53 | 85.25 |
| 80.80 | 85.71 | 82.31 | 83.37 |
| 80.47 | 84.05 | 85.54 | 91.92 |
| 72.88 | 82.67 | 87.60 | 93.32 |
| 71.74 | 82.92 | 89.73 | 89.51 |
| 70.35 | 84.46 | 83.47 | 91.57 |
| 74.82 | 83.21 | 85.21 | 88.70 |
| 86.61 | 85.85 | 90.14 | 88.31 |
| 86.65 | 80.10 | 80.12 | 90.55 |
| 86.41 | 83.38 | 82.49 | 94.93 |
| 86.85 | 83.21 | 89.79 | 92.96 |
| 82.96 | 81.26 | 88.54 | 92.29 |
| 84.75 | 75.04 | 84.59 | 94.60 |
| 76.48 | 86.28 | 92.10 | 85.33 |
| 85.69 | 84.20 | 85.65 | 94.82 |
| 79.03 | 77.81 | 89.69 | 87.86 |
| 83.05 | 72.62 | 90.10 | 84.79 |
| 79.97 | 73.73 | 86.40 | 83.56 |
| 74.86 | 85.36 | 89.67 | 82.62 |
